# *MAML2*-Rearranged Mucoepidermoid Carcinoma of the Parotid Gland: A Report in a 20-Month-Old Toddler

**DOI:** 10.1155/2022/8749836

**Published:** 2022-03-29

**Authors:** Adepitan A. Owosho, Abiodun S. Olatunji, Adewale F. Adejobi, Olawumi A. Fatusi, Toluwaniyin Okunade, Kelly Maddux, Justin Shaw, Kurt F. Summersgill

**Affiliations:** ^1^Missouri School of Dentistry and Oral Health, A.T. Still University, Kirksville, Missouri, USA; ^2^Faculty of Dentistry, Obafemi Awolowo University Teaching Hospital Complex, Ile-Ife, Nigeria; ^3^Department of Diagnostic Sciences, School of Dental Medicine, University of Pittsburgh, Pittsburgh, Pennsylvania, USA

## Abstract

Malignancies of salivary gland origin are rare in children. Mucoepidermoid carcinoma (MEC) is the most common histologic type of salivary gland neoplasm in pediatrics. We report a rare case of parotid MEC in a 20-month-old female patient. The tumor was composed of nests of epidermoid cells with nuclei appearing vesicular, pleomorphic, and hyperchromatic with an admixture of mucous cells and cystic spaces within a prominent connective tissue stroma. Immunohistochemically, the epidermoid cells showed cytokeratin 7 and P63 positivity, and mucous cells were positive for mucicarmine. Molecularly, this case was positive for *MAML2* rearrangement by FISH. To our knowledge, this is one of the youngest cases of MEC of the parotid gland reported in the English literature.

## 1. Introduction

Tumors of salivary gland origin are rare in children, representing <10% of pediatric head and neck neoplasms [1]. Mucoepidermoid carcinoma (MEC) is a malignant epithelial neoplasm composed of epidermoid, mucinous, and intermediate cells in variable proportion depending on its grade (low, intermediate, or high), forming solid and cystic patterns. MEC was first described in 1895 by Volkmann and is the most common salivary gland malignancy [2]. It makes up over half of malignant salivary gland tumors in children and is most commonly located in the parotid gland. [3]

Over 75% of MECs have been found to harbor translocations *t* (11; 19) (q21; p13), and *t* (11; 15) (q21; q26) resulting in the fusion transcripts of the *CRTC1* (*MECT1*)-*MAML2* and rarely the *CRTC3* (*MECT3*)-*MAML2* [4]. The prognostic significance of *CRTC1/3-MAML2* fusion in MEC remains questionable, as earlier studies associated it with favorable clinicopathological features and survival; however, later report showed that it can no longer be considered a prognostic marker [5–10]. We report a case of primary MEC of the parotid gland with *MAML2* rearrangement in a 20-month-old female. To our knowledge, this is one of the youngest cases of MEC of the parotid gland reported in the English literature.

## 2. Case Report

### 2.1. Clinical History

**Figure 1:**
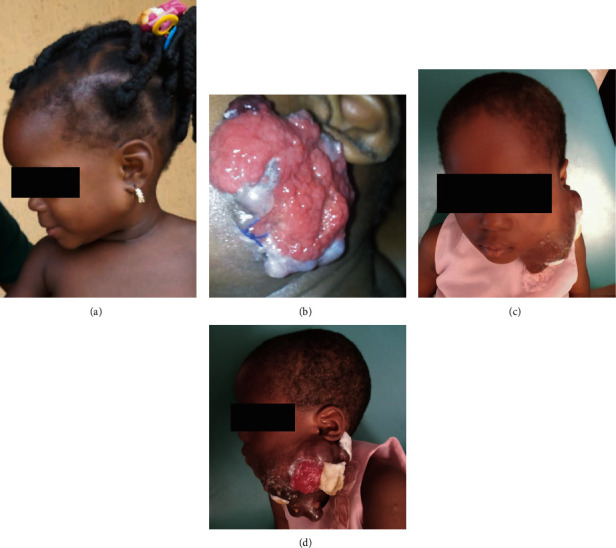
Clinical images of a female child diagnosed with a *MAML2*-rearranged mucoepidermoid carcinoma of the parotid gland. Painless parotid mass in a 1-yr 8 months old female patient (a). Few weeks after an initial incisional biopsy, an ulcerated rapidly increasing mass extending to the check area and lifting the ear (b). 2 years later, multiple ulceration sites on the 12 cm parotid mass (c, d).

**Figure 2:**
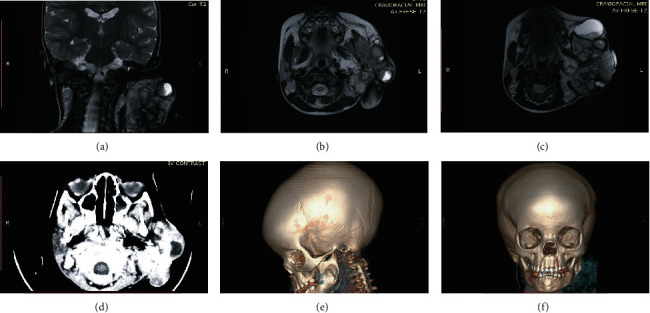
Radiologic imaging of a female child diagnosed with a *MAML2*-rearranged mucoepidermoid carcinoma of the parotid gland. MRI T2-weighted coronal and axial views (a)–(c) CT scan axial view (d), and 3D-refomatted CT scans (e, f) showing a soft tissue swelling over the left jaw region with no bony lytic or sclerotic lesion. The bony outline was preserved, with a cupping effect on the ramus of the mandible.

A left parotid painless mass was first identified in the previously healthy 1-yr 8 months old female patient (Figure 1(a)). The patient was presented to a private hospital where the mass was incompletely excised and not submitted for histopathologic evaluation. In a few weeks, the mass rapidly increased in size extensively to the check area, lifted the ear, and became ulcerated (Figure 1(b)). The patient was presented to a tertiary hospital in January 2021, where a reexcision was performed, but again, the specimen was not submitted for histopathologic evaluation. In April 2021, an incisional biopsy was performed and sent for histopathologic evaluation at Obafemi Awolowo University Teaching Hospital Complex (OAUTHC), Nigeria. In November 2021, the patient was presented to OAUTHC. At this time, the left parotid mass measured about 12 cm in the largest diameter, with multiple ulceration sites (Figures 1(c) and 1(d)). Imaging of MRI and CT with contrast of the head and neck was performed revealing a soft tissue swelling over the left jaw region with no bony lytic or sclerotic lesion; the bony outline was preserved with a cupping effect on the ramus of the mandible (Figure 2). Enlarged ipsilateral and contralateral regional lymph nodes were identified on imaging. On MRI, the tumor demonstrated variable signal intensity on T2 weighted images, ranging from hyperintense to hypointense relative to lymphoid tissue, appearing heterogeneous with cystic change (Figure 2(a)–2(c)). The CT appearance similarly showed variable mild to moderate, heterogeneous enhancement, with cystic change (Figure 2(d)).

### 2.2. Morphologic, Phenotypic, and Molecular Characteristics

Morphologic features of the tumor were of nests of epidermoid cells with nuclei appearing vesicular, pleomorphic, and hyperchromatic with an admixture of mucous cells and cystic spaces within a prominent connective tissue stroma. The immunophenotypic and molecular analyses of this tumor were performed at the University of Pittsburgh, PA. Immunohistochemical stains for CK7, P63, and mucicarmine and fluorescence in situ hybridization (FISH) for *MAML2* rearrangement were performed. Formalin-fixed, paraffin-embedded tissue was serially sectioned at 4 *μ*m intervals. An H&E stained section from the specimen was prepared and reviewed by a pathologist to identify and mark the tumor area for further analysis. A section was deparaffinized in xylene twice for 10 minutes, immersed twice in 100% ethanol, and then pretreated (1× sodium chloride, sodium citrate). The slide was digested in pepsin solution (0.75 mg/mL in 1 N HCl) followed by drying. FISH was then performed using the Dual-color breakapart FISH Probe, *MAML2* (11q21) labeled with 3′ orange and 5′ green (ZytoLight, Bremerhaven, Germany). The slide and probes were codenatured at 75°C for 5 minutes before hybridization. The slide was incubated overnight at 37°C in a humidified chamber. Posthybridization wash was performed in 2× SSC/0.3% IGEPAL for 2 minutes at 72°C. Slide was air-dried in the dark and counterstained with 4′,6-diamidino-2-phenylindole (DAPI). Analysis was performed using a Leica Biosystems (CytoVision FISH Capture and Analysis Workstation, Buffalo Grove, IL, USA). Only individual and well-delineated cells were counted; overlapping cells were excluded from the analysis. Sixty cells were scored. Normal cells without translocation show two orange/green fusion signals. Cells with the translocation show one orange/green fusion and one orange and one green signal separate (representing a translocation of *MAML2*).

Cytokeratin 7 (CK7; clone OV-TL 12/30, Dako, 1 : 200 dilution) is an automated detection using Ventana OptiView detection. For p63 (BC4A4, prediluted, Biocare Medical, Walnut Creek, CA, heat retrieval citrate buffer pH 6.0). The antibody labeling was performed with the Ventana Iview dab (2′-diaminobenzamide) detection kit (DAB, Ventana Medical Systems) as the substrate chromogen (brown).

**Figure 3:**
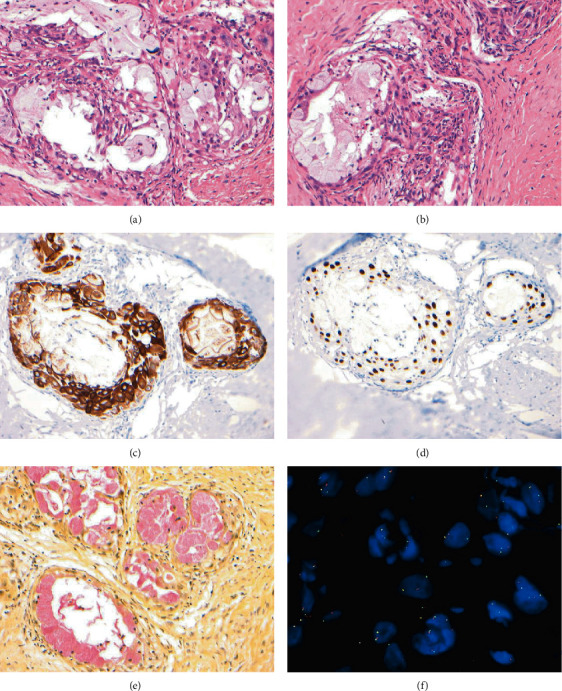
Histopathology of mucoepidermoid carcinoma of the parotid gland in a 20-month-old female patient. Morphology shows nests of epidermoid cells with nuclei appearing vesicular, pleomorphic, and hyperchromatic with an admixture of mucous cells and cystic spaces within a prominent connective tissue stroma (H&E ×200) (a, b), epidermoid cells positive for cytokeratin 7 (×200) (c), epidermoid cells positive for P63 (×200) (d), mucous cells positive for mucicarmine (×200) and (e) fluorescence in-situ hybridization for *MAML2*. Normal cells without translocation show two orange/green fusion signals. Cells with the translocation show one orange/green fusion and one orange and one green signal separate (representing a translocation of *MAML2*) (f).

**Figure 4:**
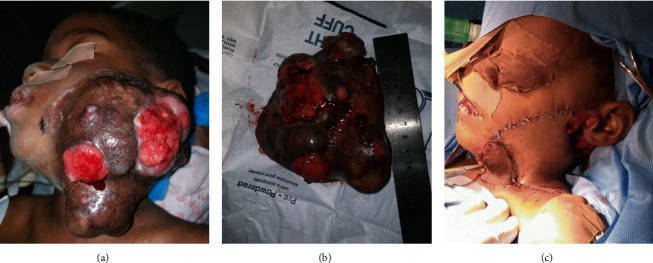
Presurgical clinical image of a female child diagnosed with a *MAML2*-rearranged mucoepidermoid carcinoma of the parotid gland (a). Massive 12 cm resected *MAML2*-rearranged mucoepidermoid carcinoma of the parotid gland (b). Reconstruction of the surgical defect with a skin flap (c).

**Figure 5:**
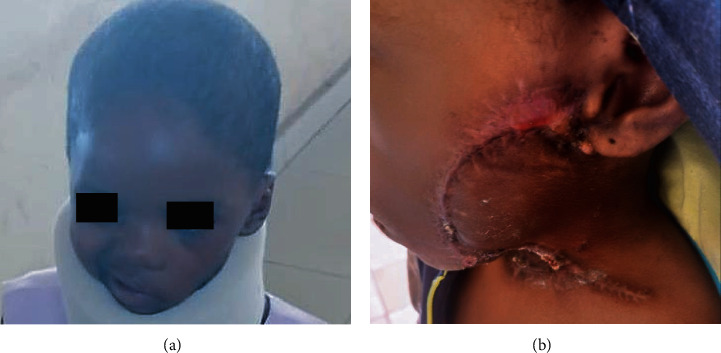
Nine weeks postsurgery clinical image of a female child diagnosed with a *MAML2*-rearranged mucoepidermoid carcinoma of the parotid gland with a left facial nerve deficit (a) and healed reconstructive skin flap (b).

The epidermoid cells were positive for CK7 and P63, and the mucous cells were positive for mucicarmine. FISH for *MAML2* rearrangement was positive with 40% of cells showing translocation of the 60 cells examined (Figure 3). The cut-off for a positive *MAML2* rearrangement is 20% of cells translocated. Additionally, an ultrasound-guided fine needle biopsy was performed on the enlarged contralateral lymph node, which was negative for tumor on histopathologic evaluation. The patient underwent total parotidectomy/surgical excision and selective neck dissection after the correction of her hematologic state (Figures 4(a)–(c)). The resected tumor and all dissected lymph nodes were evaluated histopathologically, and a diagnosis of intermediate-grade mucoepidermoid carcinoma was rendered with no lymph node involvement. At 9 weeks postsurgery follow-up visit, facial nerve deficit on the left side was apparent, and the reconstruction flap was healing properly (Figures 5(a) and 5(b)).

## 3. Discussion

The present case is distinctive because of the young age of the patient. Prior to this report, the youngest patient reported to be diagnosed with MEC of the parotid gland was a 20-month-old male child [11]. MEC is the most common epithelial or salivary gland malignancy arising in the parotid in children. MEC arises from pluripotent stem cells of the excretory ducts of salivary gland that have the potential to differentiate into squamous/epidermoid, intermediate, and mucous cells. The variable proportion of these cell types in addition to other histologic features such as necrosis, cellular/nuclear atypia, tumor invasion, and metastases determines its grade (low, intermediate, or high) by AFIP and Brandwein systems [12–14].

*CRTC1/3-MAML2* fusion has been found to be specific to MEC as a tumor of salivary gland origin and absent in other salivary gland tumors [8, 9]. *MAML2* translocation with other gene partners have been reported in neoplasms such as hidradenomas, poromas, poroid squamous cell carcinoma/porocarcinoma, metaplastic thymomas, acute lymphoid leukemia, retiform/composite hemangioendotheliomas, and rare nasopharyngeal carcinomas [15–21]. *CRTC1/3-MAML2* fusion has been described in hidradenoma, a benign adnexal neoplasm of the apical sweat gland [15]. *CRTC1/3-MAML2* fusion in MEC is more frequent in younger patients, in low/intermediate-grade and has been associated with a favorable prognosis [4, 8, 9]. A later report demonstrated that *CRTC1/3-MAML2* fusion in MEC can no longer be considered a prognostic marker [10]. However, a recent study demonstrated that *CRTC1/3-MAML2* fusion testing is a useful adjunct to histologic grading of MECs and for identifying tumors with poor prognosis with higher accuracy, thus de-escalating treatment [4]. Patients with *CRTC3-MAML2* fusion positive MEC have a better prognosis and are comparatively younger than those with *CRTC1-MAML2* fusion [8].

Many salivary gland tumors are characterized by recurrent chromosomal translocations, which may result in the upregulation of oncoproteins that can be identified by immunohistochemistry such as PLAG1 and HMGA2 in pleomorphic adenoma and carcinoma ex-pleomorphic adenoma, MYB in adenoid cystic carcinoma, NR4A3 in acinic cell carcinoma, pan-Trk in secretory carcinoma, and MAML2 in mucoepidermoid carcinoma [22–29]. These immunohistochemical stains can serve as ancillary diagnostic adjuncts.

The mainstay of management of pediatric salivary gland cancers is by surgical resection. A recent systematic review on survival of 698 children treated for salivary gland cancers revealed that 95% of the children were treated with primarily surgery, 65% were treated with surgery alone, and about a quarter received adjuvant radiotherapy with 93% of the children alive as at the last follow-up [3]. Another review of 73 pediatric MEC patients treated with a 10-year disease specific survival rate was 88% and showed that all patients received surgery, 15 patients underwent adjuvant radiotherapy, and 4 patients received adjuvant chemotherapy [30]. Prognosis for children with MEC of the parotid gland is good.

In conclusion, this report describes a rare case of *MAML2*-rearranged mucoepidermoid carcinoma of the parotid gland in a 20-month-old female child. The delay in the management of this patient highlights problems in the healthcare system of a developing country such as poor referral system, lack of medical insurance for all children and social services, patient (parent) education, and cultural beliefs. This case highlights the importance of histopathological evaluation of biopsy specimens as a useful tool in the early and accurate diagnosis of head and neck malignancies. The failure in submission of the biopsy specimens on two occasions for histopathologic evaluation contributed to the delay in management of this patient.

## Data Availability

No data were used to support this report.
